# ICP Materials Trends in Corrosion, Soiling and Air Pollution (1987–2014)

**DOI:** 10.3390/ma10080969

**Published:** 2017-08-19

**Authors:** Johan Tidblad, Kateřina Kreislová, Markus Faller, Daniel de la Fuente, Tim Yates, Aurélie Verney-Carron, Terje Grøntoft, Andrew Gordon, Ulrik Hans

**Affiliations:** 1 Swerea KIMAB, Dept Corrosion, 164 07 Kista, Sweden; andrew.gordon@swerea.se; 2 Svuom Ltd., 17000 Prague, Czech Republic; kreislova@svuom.cz; 3 Empa, Materials Science and Technology, 8600 Dübendorf, Switzerland; markus.faller@empa.ch (M.F.); ulrik.hans@empa.ch (U.H.); 4 CENIM—National Centre for Metallurgical Research, 28040 Madrid, Spain; delafuente@cenim.csic.es; 5 BRE—Building Research Establishment Ltd., Watford WD25 9XX, UK; yatest@bre.co.uk; 6 LISA (Laboratoire Interuniversitaire des Systèmes Atmosphériques), UMR 7583 CNRS/UPEC/UPD, 94010 Creteil, France; aurelie.verney@lisa.u-pec.fr; 7 NILU—Norwegian Institute for Air Research, 2027 Kjeller, Norway; terje.grontoft@nilu.no

**Keywords:** atmospheric corrosion, soiling, pollution, carbon steel, weathering steel, zinc, copper, aluminium, limestone, glass

## Abstract

Results from the international cooperative programme on effects on materials including historic and cultural monuments are presented from the period 1987–2014 and include pollution data (SO_2_, NO_2_, O_3_, HNO_3_ and PM_10_), corrosion data (carbon steel, weathering steel, zinc, copper, aluminium and limestone) and data on the soiling of modern glass for nineteen industrial, urban and rural test sites in Europe. Both one-year and four-year corrosion data are presented. Corrosion and pollution have decreased significantly and a shift in the magnitude is generally observed around 1997: from a sharp decrease to a more modest decrease or to a constant level without any decrease. SO_2_ levels, carbon steel and copper corrosion have decreased even after 1997, which is more pronounced in urban areas, while corrosion of the other materials shows no decrease after 1997, when looking at one-year values. When looking at four-year values, however, there is a significant decrease after 1997 for zinc, which is not evident when looking at the one-year values. This paper also presents results on corrosion kinetics by comparison of one- and four-year values. For carbon steel and copper, kinetics is relatively independent of sites while other materials, especially zinc, show substantial variation in kinetics for the first four years, which needs to be considered when producing new and possibly improved models for corrosion.

## 1. Introduction

“ICP Materials” or the “International co-operative programme on effects on materials including historic and cultural monuments” is an international project that has been run since the 1980’s (www.corr-institute.se/icp-materials). The program started, together with other international cooperative programmes (ICP’s) on effects on ecosystem and health, as a reaction to environmental problems faced in Europe and North America. The science produced within ICP Materials is in support of the Convention on Long-range Transboundary Air Pollution (LRTAP Convention), within the United Nations Economic Commission for Europe.

Over the years, almost eighty reports have been issued with results of the program, and many different scientific publications. Some scientific publications also reflect important environmental concerns. The first main publication of results was published in 2001 “Dose-response functions on dry and wet acid deposition effects after 8 years of exposure”, and included results from the period 1987–1995 and empirical relations on how to calculate atmospheric corrosion attack based on environmental parameters [1]. The main environmental concern was acid rain and acidifying pollutants. The next important publication (2007) was named “Dose-response functions for the multi-pollutant situation” [2]. With decreasing levels of sulphur dioxide, it was realized that more complicated expressions were needed to successfully predict atmospheric corrosion, including other pollutants, such as nitric acid and particulate matter. During this period, climate change started to be high on the agenda and it was realized that dose–response functions from international exposure programs could also be used to assess the possible impact of long term changes in climate on corrosion. “Atmospheric corrosion of metals in 2010–2039 and 2070–2099” described this procedure, and was published in 2007 [3]. Finally, in 2012, the latest main publication from ICP Materials was released “ICP Materials Celebrates 25 Years of Research” [4]. This open source publication had the purpose of giving a complete metadata description of all the data available from the program, including citations to main data sources and publications.

ICP Materials is not the only international exposure program; other important programs include, for example, National Acid Precipitation Assessment Program (NAPAP) in the US [5], ISO CORRAG worldwide [6], Ibero-American Map of Atmospheric Corrosiveness (MICAT) in Ibero-American countries [7] and the corrosion network (CORNET) of the regional air pollution in developing countries (RAPIDC) in Asia/Africa [8]. However, ICP Materials is unique in its persistence, which has enabled it to perform exposures for thirty years (1987–2017) and there are currently plans for continuation of the program up to at least 2021.

The purpose of this publication is twofold. The first, and maybe most important, is to give an overview of the data, not only in normal publication format, but also to release a comprehensive database on corrosion, soiling and air pollution, available for download. This will enable independent researchers to quickly access the data, to check conclusions and to perform their own analysis. Naturally, this database does not include all data from the program, but this is the first time data from ICP Materials are released in this format, and releases will hopefully continue. The second is to provide an overview of the main trends in corrosion, soiling and pollution during the whole period (1987–2014) and during recent years. This gives the background for the decisions currently taken on how to develop the program in the coming years, which will include an increased focus on soiling of materials, mainly as a result of particulate matter deposition.

## 2. Results

ICP Materials exposure sites have changed over the years. In this publication, only data from the following sites are considered. The selection was made from sites that are currently active and have more than just a few years of data. Selected sites include three industrial sites, nine urban sites and seven rural sites, in total nineteen sites:

- Industrial sites: Kopisty, Bottrop and Katowice;
- Urban sites: Prague, Rome, Milan, Venice, Oslo, Stockholm, Madrid, Paris and Berlin; and
- Rural sites: Casaccia, Birkenes, Aspvreten, Toledo, Lahemaa, Svanvik and Chaumont.

Most of these sites were included from the beginning of the program (1987), except Katowice, Paris, Berlin, Svanvik and Chaumont, which were introduced later (1995–2000). It should be noted that these labels “Paris”, “Berlin”, etc. indicate that they are located in these cities. The values of corrosion, pollution and soiling at these sites should however not be considered representative of these cities, since the variation of corrosion and pollution within a city can be substantial.

Environmental parameters included in this publication are the pollutants SO_2_, NO_2_, O_3_, HNO_3_ and PM_10_. Materials included in this publication are carbon steel, weathering steel, zinc, copper, aluminium, limestone and modern glass.

Before presenting the results, a special note on exposure periods is needed. All exposures performed in ICP Materials so far have started in the fall, usually in October, and then lasted for one year or several years, also ending in the fall. In this paper, all exposure periods are labelled with the start year so that, for example, “1987” in reality is a short hand notation for a one-year exposure between the fall of 1987 to the fall of 1988, and “2011–2014” is a short hand notation for a four-year exposure between the fall of 2011 and the fall of 2015.

### 2.1. Trends in Air Pollution

**Figure 1:**
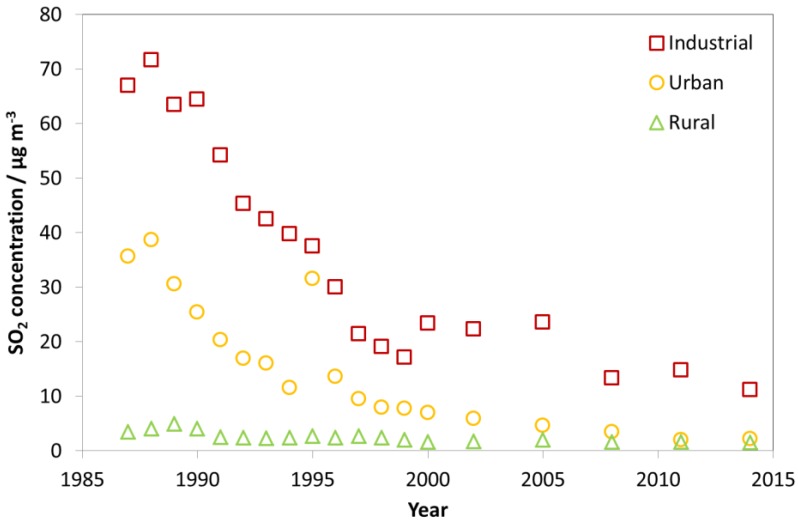
Average SO_2_ concentration at industrial, urban and rural sites for individual years (1987–2014).

The concentrations of the pollutants SO_2_, NO_2_, O_3_, HNO_3_ and PM_10_ in air (µg m^−3^), as measured at the ICP Materials sites, are included in the description of trends in air pollution. Figure 1 shows that the average of the SO_2_ concentrations at the industrial sites was considerably higher than for the urban and rural sites, in all the measurement years since 1987, except in 1995. In 1995, the value at the urban sites was much higher than in the measurement years just before and after, and nearly as high as for the industrial sites. The reason for this is technical and coincidental. It is due to a change of sites in the measurement programme, with only one urban site remaining in 1995, before a set of the former, and one new, urban sites were again included in 1996. This highlights a difficulty presenting averages and is the reason why this paper also presents trends for individual sites to illustrate characteristic trends. Until 1997, the average SO_2_ concentration measured at the industrial sites was above 30 µg/m^3^. The average SO_2_ concentration measured at the urban sites was close to the average values measured for all the sites, in all years. By 1997, it had been reduced to below 10 µg/m^3^, and, by 2005, to below 5 µg/m^3^. The average SO_2_ concentration measured at the rural sites was below 5 µg/m^3^ in all the years.

**Figure 2:**
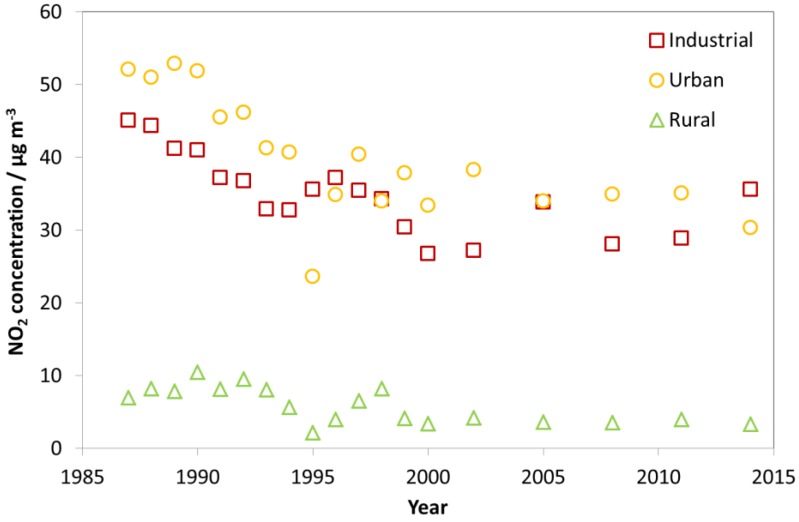
Average NO_2_ concentration at industrial, urban and rural sites for individual years (1987–2014).

Figure 2 shows a quite different situation for NO_2_ as compared to SO_2_. The average NO_2_ concentration has not changed much since 1987. Some decreases in concentrations were measured until approximately 2000, with little change thereafter, except some possible increase at the industrial sites. In contradiction to the situation for SO_2_, the average concentration of NO_2_ at the urban sites was higher than at the industrial sites in nearly all years, with the coincidental main exception being 1995, when only one urban site was included in the average. The higher average concentrations of NO_2_ at the urban sites may have been due to more emissions from traffic and domestic heating than at the industrial sites. The average concentration of NO_2_ at the rural sites was generally below 10 µg/m^−3^, but with somewhat higher concentrations and more variation between years before 2000.

**Figure 3:**
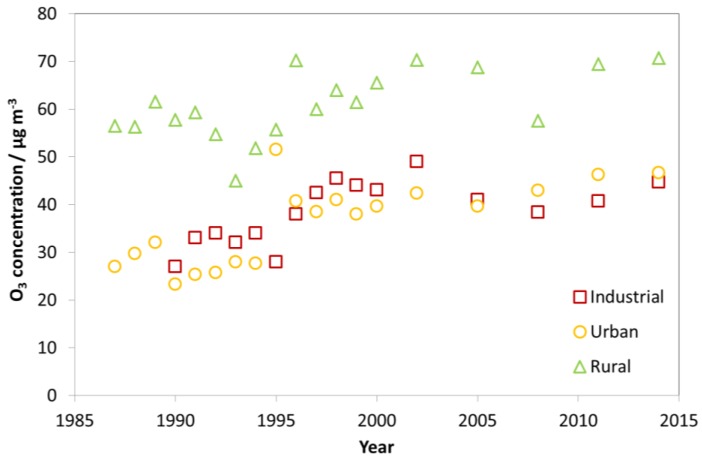
Average O_3_ concentration at industrial, urban and rural sites for individual years (1987–2014).

Figure 3 shows a quite different situation for O_3_ as compared to SO_2_ and NO_2_. The trends for the average concentration of O_3_ measured for the sites were slightly positive, and slightly more so from 1987 to about 2000 than from 2000 to 2014. The average concentration at the rural sites was always significantly higher than that at the urban and industrial sites. The difference between the average concentration at the urban and industrial sites was always small, with alternating ranking between them. In 1995, there was only one urban site in the calculated average, due to changing of sites in the programme, which explains the coincidental high “average” value in this year.

**Figure 4:**
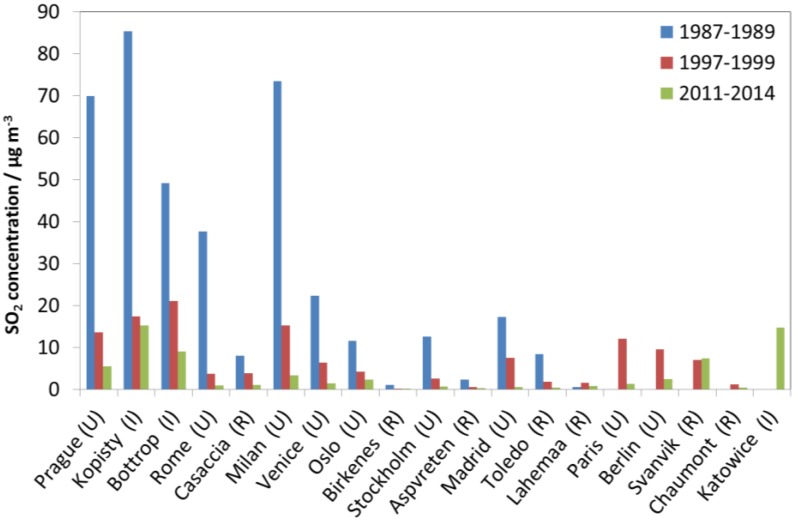
SO_2_ concentration at individual sites based on averages for three selected periods, 1987–1989, 1997–1999 and 2011–2014.

**Figure 5:**
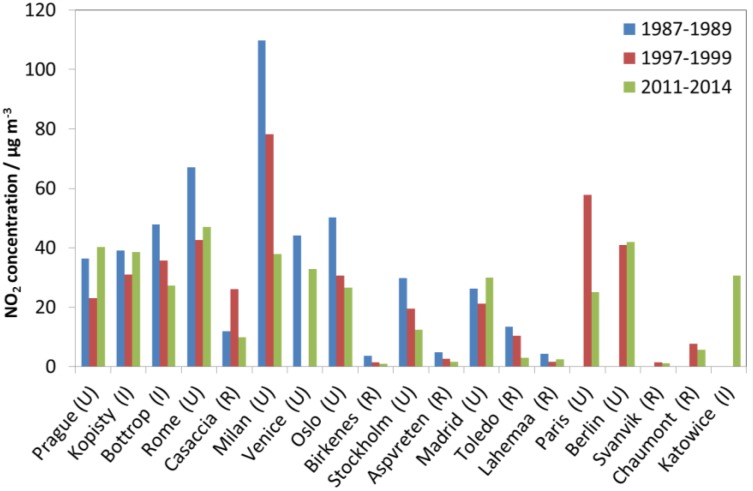
NO_2_ concentration at individual sites based on averages for three selected periods, 1987–1989, 1997–1999 and 2011–2014.

**Figure 6:**
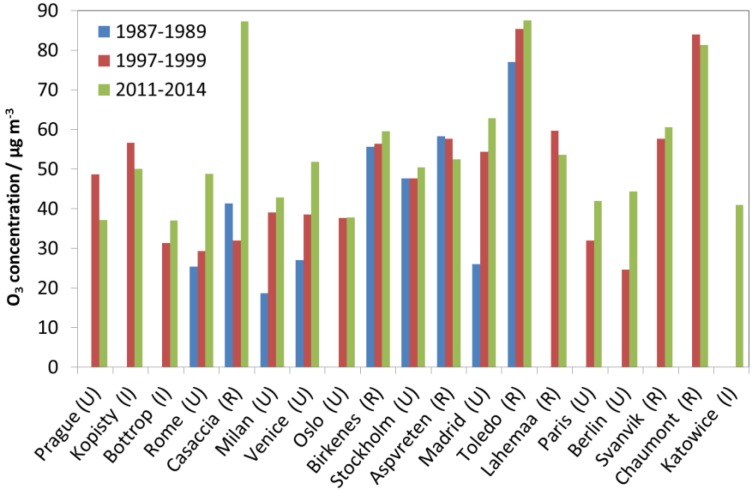
O_3_ concentration at individual sites based on averages for three selected periods, 1987–1989, 1997–1999 and 2011–2014.

Looking at Figure 1, Figure 2 and Figure 3, there is a change in the trend of air pollution around 1997–1999 where a steep change is replaced by a more modest change (decrease for SO_2_ and NO_2_ and increase for O_3_). Therefore, a compact way of summarising the data from all sites is to present diagrams with averages for the three periods 1987–1989, 1997–1999 and 2011–2014. This will more or less summarise the whole trend for all sites as well as give the opportunity to compare changes for different periods (1987–1989 vs. 1997–1999, 1987–1989 vs. 2011–2014, and 1997–1999 vs. 2011–2014). Figure 4, Figure 5 and Figure 6 show this type of diagram for SO_2_, NO_2_ and O_3_, respectively.

Figure 4 shows that the decrease of SO_2_ at the industrial sites for the whole period of measurements, from 1987–1989 to 2011–2014, was dominated by a decrease in Kopisty, but from 1997–1999 by a decrease in Bottrop. The decrease at the urban sites from 1987–1988 to 2011–2014 was dominated by a decrease in Milan and Prague, with somewhat less decrease in Rome, Venice and Madrid. Since 1997–1999, the decrease was larger in Prague than in Milan and the ranking of the other sites also changed. The decreases at the new sites in Paris and Berlin were higher than in Madrid and Venice. The few measurement values for Rome showed a situation there after 1997–1999 was more similar to that of the low values in Stockholm and Oslo. It can however be noted that a relatively high SO_2_ concentration as compared to previous years, of 4.4 µg/m^3^, was measured in Oslo in 2014. This points to the need, still, for measurements of SO_2_ in the present situation with generally low concentrations, and for attention to possible new or reappearing emission sources. Some notable decreases in SO_2_ since 1987–1989 were also observed at the rural sites.

Figure 5 shows a decrease in the NO_2_ concentrations measured at most of the sites since 1987–1989, with an overall correspondence to the decrease for SO_2_, with the highest decrease in Milan, but with some noteworthy exceptions. Foremost among them, in Prague and Madrid, there was a small increase in the concentration measured for NO_2_ since 1987–1989 and a higher increase since 1997–1999. In both cases, NO_2_ was observed to decrease until 2000 and increase thereafter. During 1997–1999, NO_2_ was measured at four more sites, Paris, Berlin, Svanvik and Chaumont, than during 1987–1989. Measurements at the site Katowice started in 2000. In addition to the increase in NO_2_ in Prague and Madrid, the concentration measured for NO_2_ has also increased in Kopisty and Rome since 1997–1999. The reasons for these increases were high values for 2014 in Kopisty and 2011 in Rome.

The decreases in measured NO_2_ in Stockholm and Oslo since 1987–1989, and in Casaccia and Toledo since 1997–1999, as compared to the other sites, were relatively larger for NO_2_ than for SO_2_. Among the new sites included from 1997–1999 to 2011–2014, a large decrease in NO_2_ was measured in Paris.

Notable decrease in NO_2_ was measured for the rural site of Toledo since 1992, and since 1997–1999. A general trend of slightly decreasing NO_2_ was measured at the rural sites since 1987–1989 and 1997–1999, but with some high values for some years and variation in values for some sites, which could influence trend calculations. Most notably, at the site Casaccia, much higher values were measured in 1997 and 1998 than in other years, giving increase in concentration to 1997–1999 and decrease thereafter.

Figure 6 shows quite large increases in O_3_ concentrations at the Italian sites Casaccia, Venice, Milan and Rome, and Spanish sites Madrid and Toledo from 1987–1989 to 2011-2014. The increases were mainly due to low start values in 1987–1989, but also partly due to high values during 2011–2014 in Casaccia, Toledo and Madrid.

At the sites in Rome and Casaccia, lower values were measured in the mid-1990s, and then considerable increases were measured towards 2011–2014. Significant increases were also observed from 1997–1999 to 2011–2014 for some sites outside of Italy and Spain, for which O_3_ measurement results then had become available: Berlin, Paris and Bottrop. The changes in O_3_ showed no clear correlation with changes in NO_2_ and SO_2_. The largest variation and overall increase in O_3_ concentration was measured for the rural site Casaccia. Except for this site, the sites with the largest increases were all urban sites. At the other rural sites, and the urban sites Stockholm and Oslo, small increases or decreases were measured. Exceptions from this were the two Czech sites, the industrial site Kopisty and the urban site Prague, where large decreases were measured from 1997–1999 to 2011–2014.

In contrast to SO_2_, NO_2_ and O_3_, measurements of HNO_3_ and PM_10_ were not started in the programme until 2002. Furthermore, PM_10_ is not a mandatory parameter to measure. Therefore, the data for HNO_3_ and PM_10_ do not permit evaluation of long-term trends in the same manner as for SO_2_, NO_2_ and O_3_. Instead, data for individual sites are presented, in a similar way as for SO_2_, NO_2_ and O_3_, but using only two periods: 2002–2005 and 2011–2014. With measurement results for PM_10_ and HNO_3_ for only four to five years, any interpretation of trends should be made with caution.

**Figure 7:**
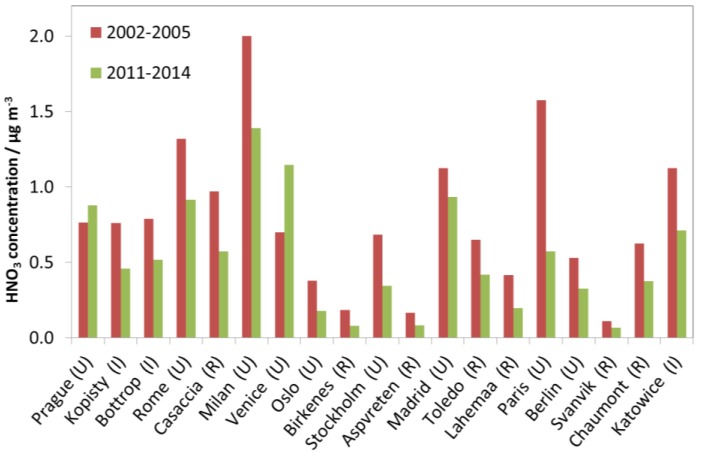
HNO_3_ concentration at individual sites based on averages for two selected periods, 2002–2005 and 2011–2014.

Figure 7 shows the concentration of gaseous nitric acid (HNO_3_). Comparing the two periods, the average concentration measured for most sites has decreased. There is considerable variation between the values for the industrial, urban and rural sites, with higher values measured at some rural sites than at the urban and industrial sites. The highest values were measured at the urban sites Paris, Milan, Venice, Rome and Madrid, and the industrial site Katowice. Notably, low values were measured at the rural sites Svanvik, Aspvreten and Birkenes, and at the urban sites Oslo, Stockholm and Berlin. Details on the measurements of nitric acid have been presented elsewhere [9].

**Figure 8:**
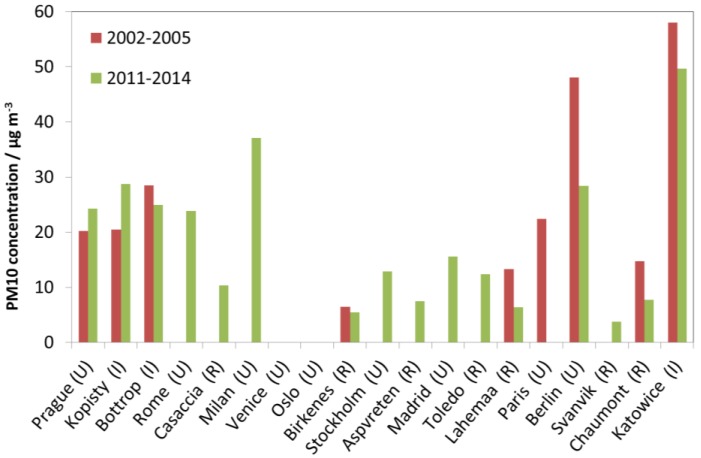
PM_10_ concentration at individual sites based on averages for two selected periods, 2002–2005 and 2011–2014.

Figure 8 shows the concentration in air of particulate matter with aerodynamic diameter smaller than 10 µm (PM_10_). It is not possible to make any general conclusions regarding trends based on this limited dataset. Among the urban sites, notably low values were measured at the sites of Stockholm and Madrid. Among the rural sites, the highest concentrations measured overall were in Toledo, and the lowest concentrations were measured in Birkenes and Svanvik. Some sites showed notable trends of decreasing PM_10_, including the urban site Berlin and the rural sites Chaumont and Lahemaa.

In summary, the trends in the changes of the average concentration of SO_2_, NO_2_ and O_3_ for the ICP sites were all stronger in the first phase of the programme, from 1987 to about 2000, than thereafter. This was very apparent for SO_2_, clearly seen for NO_2_ for which there were no apparent trend after 2000, and slightly apparent for the positive trend for O_3_.

From about 2000 to 2014, a trend of gradual decrease in the SO_2_ concentration was measured at all sites, except Svanvik where a small increase was measured. The Svanvik site is located only 6 km away from the considerable SO_2_ source of the nickel plant in the town of Nikel, Russia. There was no general trend in the measured concentration of NO_2_ since 1997–1999, but for more of the sites decreases were measured than increases, with the largest changes in the measured concentrations being the decreases at the sites Milan (−40 µg/m^3^) and Paris (−33 µg/m^3^). Other sites where considerable decreases in NO_2_ were measured were Casaccia, Bottrop, Toledo and Stockholm. Considerable increases in NO_2_ were measured at the sites Prague, Madrid, Kopisty and Rome. A slight positive trend was measured for O_3_ from 1997–1999 to 2011–2014 for nearly all the urban sites, except Prague, and for the rural site Casaccia. Since 2002, a clear and continuous decreasing trend in the concentration of HNO_3_ was measured at all the industrial sites, at the urban sites Paris and Milan, and at the rural sites Casaccia and Toledo. Since 2002, a clear decreasing trend in PM_10_ was measured at the Berlin and Chaumont sites.

Taken together, for air pollution, the largest recent (since about 2000) decreases in concentration at ICP sites were measured in Milan and Paris, then Bottrop and then the other urban and industrial sites. However, considerable increases in NO_2_ were measured in Prague, Madrid and Kopisty, and considerable increases in O_3_ in Rome and Berlin. The changes for the rural sites were minor compared to the industrial and urban sites, but with notable decrease of NO_2_ in Toledo, and Casaccia, where the variation in NO_2_ between years and the increase in O_3_ was the largest.

### 2.2. Trends in Carbon Steel Corrosion

**Figure 9:**
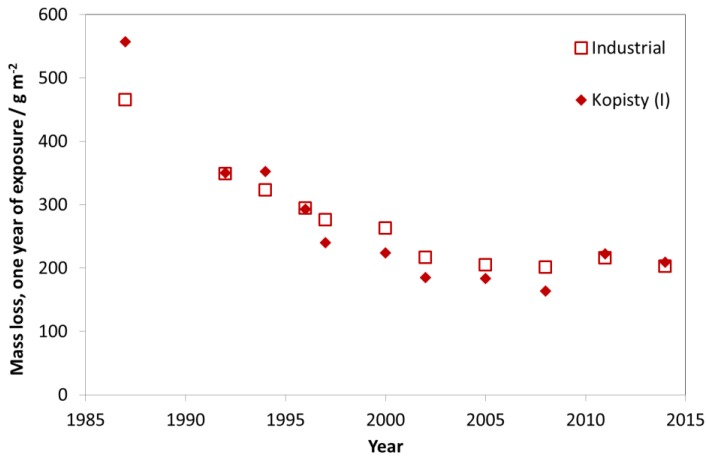
Carbon steel corrosion, average of industrial sites and the industrial site Kopisty.

Figure 9 shows the average mass loss of unalloyed carbon steel (C < 0.2%, P < 0.07%, S < 0.05%, Cu < 0.07%) for one-year exposures for industrial test sites with one selected individual site, Kopisty. The corrosion decreased significantly between 1987 and 1997. The corrosion then remained on a level around 240 g/m^2^ for the first exposure year, corresponding to a 50% decrease compared to the original value. In the last 25 years, the pollution at Kopisty reduced tremendously because of the decline of heavy industry. ISO 9223 corrosivity category C3 ranges from 200 to 400 g/m^2^. With a recent value of 210 g/m^2^ for the first year of exposure, this industrial test site has changed its corrosivity category during 1987–2014 from C4 to low C3.

**Figure 10:**
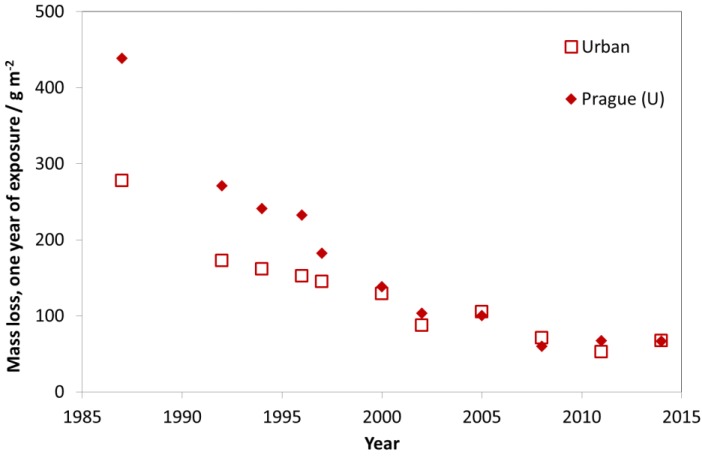
Carbon steel corrosion at urban sites and the urban site Prague.

In Figure 10, the trend of carbon steel corrosion is shown for the nine urban test sites with Prague as a typical example. As for the industrial sites, there is a strong decrease of mass loss between the exposures in 1987 and 1997, but also a small reduction of mass loss in the following periods.

**Figure 11:**
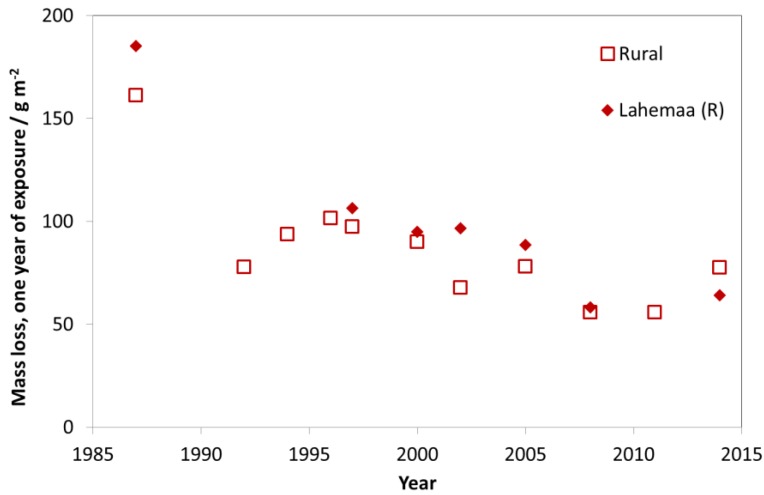
Carbon steel corrosion at rural sites and the rural site Lahemaa.

The trend for rural test sites is similar to the trends of industrial and urban test sites. For rural test sites, a slight decrease of mass loss values was found (see Figure 11). Today, the urban test sites (C4 and C3, according to ISO 9223) are tending towards C2 with values typical for former rural atmospheres. This shows that labels such as “industrial”, “urban”, “rural” and “marine” can be useful for indicating the type of pollution (dominated by SO_2_, NO_2_, O_3_ and chloride) but that they are not at all useful for classifying levels of corrosivity in a quantitative way.

Two repeated four-year exposures were performed, during 1997–2000 and 2011–2014. As for the one-year exposures, all test sites showed a decrease of corrosion values.

**Figure 12:**
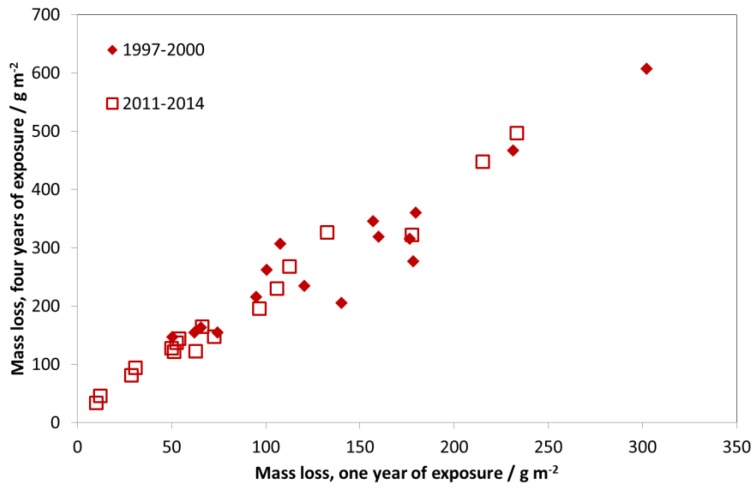
Carbon steel corrosion: one-year vs. four-year exposures for two different periods, 1997–2000 and 2011–2014. The one-year corrosion values are calculated as averages of two one-year exposures for the years 1997/2000 and 2011/2014, corresponding to the first and fourth years of the corresponding four-year period.

As an exposure was not performed during 1987–1990, a comparison of these two exposures does not show such a large decrease in corrosion rates, since the period of maximal reduction of air pollution (1987–1997) is not included. Figure 12 shows the mass loss after four years of exposure vs. mass loss after one year of exposure for two different exposure periods, 1997–2000 and 2011–2014. The clear relationship between one- and four-year periods shows that it is equivalent to show trends in corrosion based on four-year exposures instead of one-year exposures. The advantage of using four-year values for showing trends in corrosion is that it is less sensitive to year-to-year variation in climatic parameters (temperature, relative humidity, and precipitation). The higher corrosion values also make it easier to identify significant trends for individual sites. Note that in Figure 12, the relationship between one- and four-year values is practically 1:2, corresponding to a square-root kinetics for carbon steel during the first four years of exposure.

**Figure 13:**
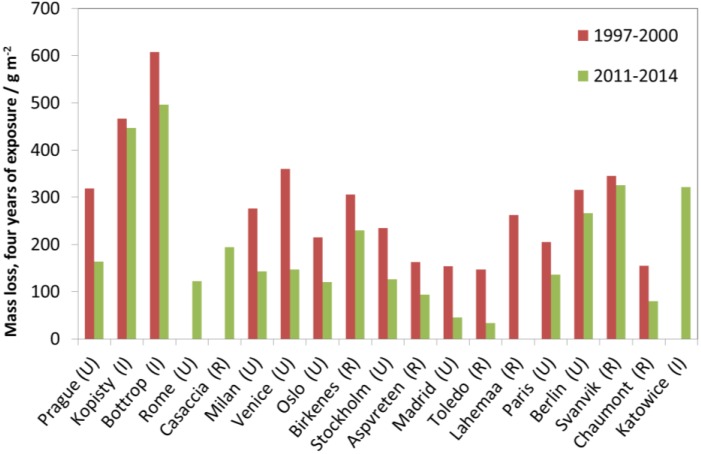
Carbon steel corrosion: four-year exposures at individual sites for two exposure periods, 1997–2000 and 2011–2014.

Figure 13 shows trends of four-year corrosion at the test sites and that corrosion has decreased at all sites corresponding to average levels for 2011–2014 of about 60% of those for 1997–2000.

### 2.3. Trends in Weathering Steel Corrosion

**Figure 14:**
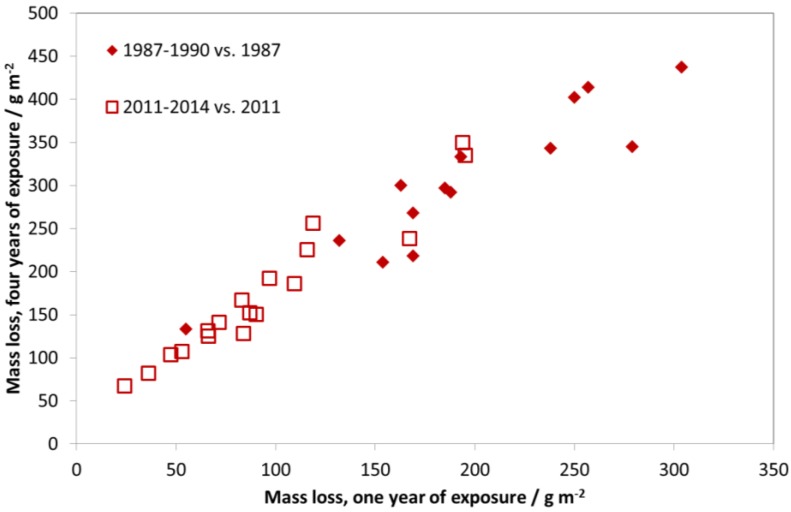
Weathering steel corrosion: one-year vs. four-year exposures for two different periods, 1987–1990 and 2011–2014. The one-year corrosion values are only available for the first years of the four-year periods, 1987 and 2011.

The exposed weathering steel (C < 0.12%, Mn 0.3%–0.8%, Si 0.25%–0.7%, P 0.07%–0.15%, S < 0.04%, Cr 0.5%–1.2%, Ni 0.3%–0.6%, Cu 0.3%–0.55%, Al < 0.01%) is a low-alloyed steel with improved corrosion performance in polluted areas in unsheltered positions, especially after longer exposure times. Only two one-year exposures have been carried out, 1987 and 2011, and two four-year exposures, 1987–1990 and 2011–2014. Figure 14 shows the mass loss after four years of exposure vs. mass loss after one year of exposure for the two different pairs. The relationship between one- and four-year periods is not as clear as for carbon steel.

At lower corrosion values, the one- to four-year corrosion relationship is closer to 1:2, as for carbon steel, but, at higher corrosion values, the one- to four-year corrosion relationship approaches about 2:3, confirming the improved performance of weathering steel after longer exposure periods.

**Figure 15:**
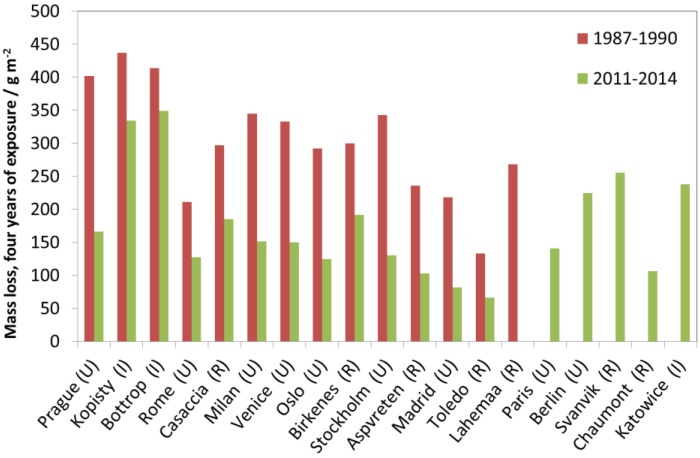
Weathering steel corrosion: four-year exposures at individual sites for two exposure, periods 1987–1990 and 2011–2014.

Figure 15 shows the four-year values for all sites. A general, significant decrease can be observed, and the corrosion was reduced by about 50%. As can be observed, the highest decreases correspond to urban sites, i.e., Madrid, Stockholm, Milan, Prague and Oslo, whereas the lowest correspond to both industrial sites, i.e., Kopisty and Bottrop. At the rural sites and some urban sites, an intermediate corrosion reduction was obtained. Similar to the case of carbon steel and other metals, the main reason for this corrosion diminishing is the general decrease in SO_2_ levels, especially from 1987 to about 2000.

### 2.4. Trends in Zinc Corrosion

Zinc (>98.5%) with two different kinds of surface preparation, ground and glass blasted, has been exposed in the programme; the ground from the beginning of the programme, from 1987, which was then replaced with the blasted from 1997. Simultaneous exposures were performed in 2000 and 2008. The glass blasted zinc has a rougher surface leading to, at least initially, higher corrosion loss values.

**Figure 16:**
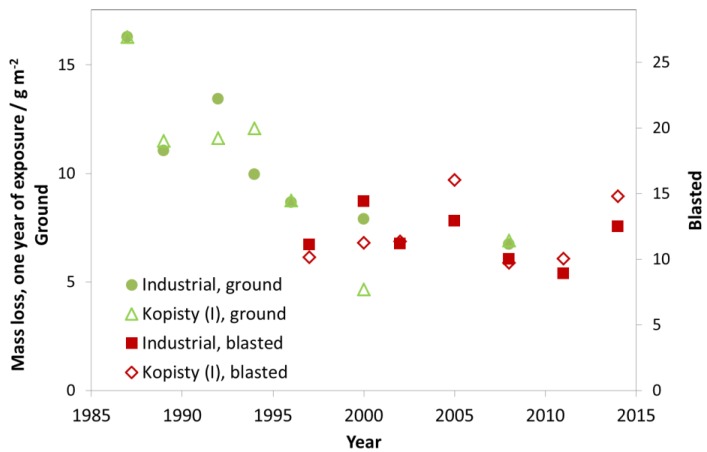
Zinc corrosion, ground and blasted at industrial sites and the industrial site Kopisty.

As can be seen in Figure 16, the mass loss after one year for the investigated industrial test sites decreased significantly in the period 1987–1997. From 1997, the value then remained at a constant level (around 10 g/m^2^) for the first exposure year (blasted zinc). The mass loss values for Kopisty are shown as an example for an industrial site, where the ISO 9223 corrosivity category changed from C4 to C3 (ISO 9223 corrosivity category C3 ranges from 5 to 15 g/m^2^).

**Figure 17:**
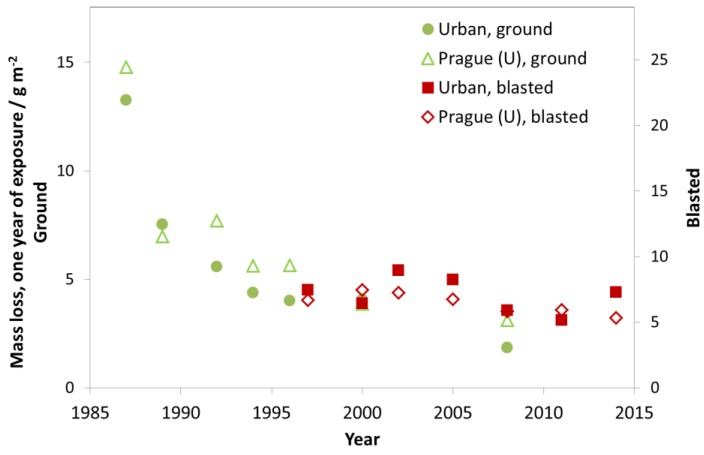
Zinc corrosion, ground and blasted at urban sites and the urban site Prague.

In Figure 17, the trend is shown for the nine investigated urban test sites with Prague as a typical example. As for the industrial sites, there is a strong decrease of mass loss between exposure periods 1987–1988 and 1997–1998, but also a small reduction of mass loss in the following periods. Today, the corrosivity category for the urban test sites (C3) is starting to approach C2 with values comparable to those in rural atmospheres.

**Figure 18:**
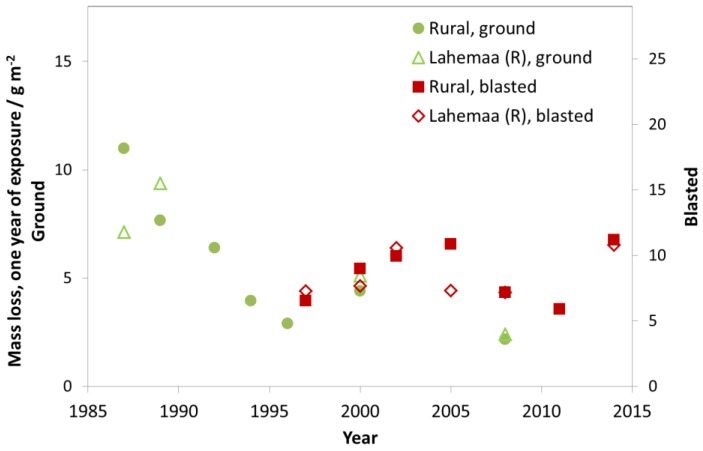
Zinc corrosion, ground and blasted at rural sites and the rural site Lahemaa.

The trend for rural test sites is slightly less distinct compared to industrial and urban test sites. There is a higher fluctuation of mass loss values from year to year. For some rural test sites, a slight decrease of mass loss values can be found but there are other sites, such as Lahemaa (Estonia), with no clear trend (Figure 18). Higher mass loss values were sometimes measured at rural sites compared to urban sites. Overall, the mass loss values (blasted) at most sites now range 5–10 g/m^2^. These values correspond to a thickness reduction of 0.7–1.4 µm, calculated based on the density of zinc 7.14 g cm^−3^ (thickness reduction in µm = mass loss in g m^−2^/7.14).

**Figure 19:**
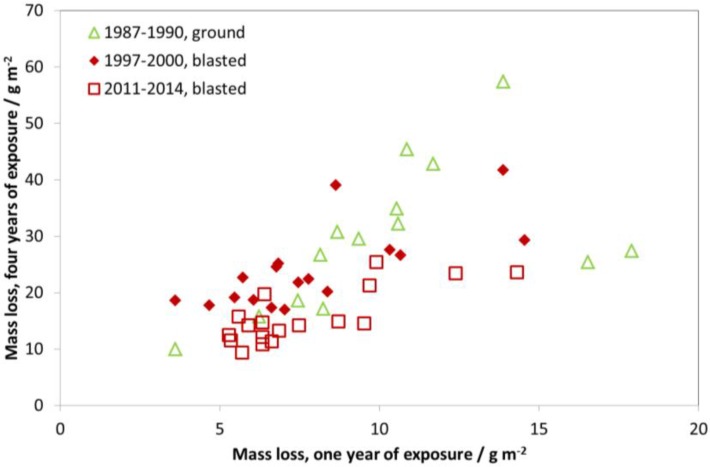
Zinc corrosion: one-year vs. four-year exposures for three different periods, 1987–1990 (ground zinc), 1997–2000 (blasted) and 2011–2014 (blasted). The one-year corrosion values are calculated as averages of two one-year exposures for the years 1987/1990, 1997/2000 and 2011/2014, respectively.

Three repeated four-year exposures, starting from 1987, were undertaken with zinc samples in unsheltered exposure at different test sites. The first exposure period (1987–1988) was with ground surface condition and the other two with blasted surface condition. Figure 19 shows a comparison between four- and one-year corrosion, similar to for carbon steel (Figure 12) and weathering steel (Figure 14). In contrast to the other materials, there is a systematic difference when looking at the different periods. For ground zinc, the relationship between the mass loss of the one- and four-year samples is about 1:4, corresponding to a line going through the point 15 g m^−2^ (one year of exposure) and 60 g m^−2^ (four years of exposure), except for two sites. If the relationship between one- and four-year data were exactly 1:4, this would correspond to a linear development of corrosion with time, i.e., linear kinetics. For blasted zinc, the four-year values are lower than what would be expected from linear kinetics, indicating that the high corrosion values resulting from blasting as opposed to ground is an initial phenomenon most prominent after one year of exposure.

**Figure 20:**
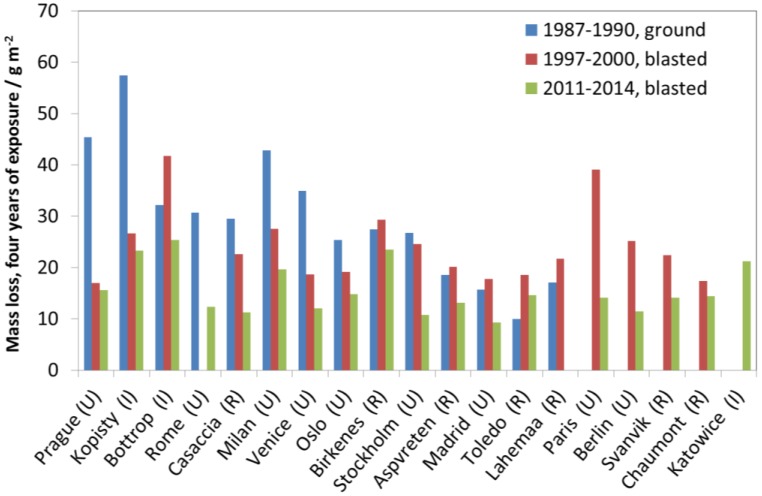
Zinc corrosion; four-year exposures at individual sites for three exposure periods, 1987–1990 (ground), 1997–2000 (blasted) and 2011–2014 (blasted).

Figure 20 shows all four-year data for the individual sites. The first value (1987–1990) is not directly comparable to the two later values due to the different surface treatment. However, it is not possible to make a correction factor since, parallel four-year exposures have not been performed. Furthermore, results presented in Figure 19 show that it is not possible to use a comparison of one-year values to derive a correction value for four-year samples. Nevertheless, it has some merit to present ground zinc (uncorrected) in the same diagram as blasted zinc, even if the decrease in corrosion between 1987 and 1990 and the other periods will be underestimated in this way.

An example of a strong decreasing trend is the test site Kopisty. The mass loss at the first exposure period (1987–1990) was around 60 g/m^2^ during four years (which means an average yearly corrosion rate of 15 g/(m^2^ year) or 2.1 µm/year). This value decreased to 23 g/m^2^ for the third exposure period (2011–2014). The corrosion rate at this test site now lies at 5.8 g/(m^2^ year) or 0.8 µm/a and can now be characterised as C3. This means a reduction of the corrosion rate of 66% from the value of the first exposure period. In practical terms, this means that a galvanized steel structure with a typical zinc layer thickness of 80 µm would previously have shown red rust (1987) after approximately 30 years, while it would now take 100 years. It should be noted that it is difficult to accurately estimate the long-term corrosion rate based on these values, especially considering the results presented in Figure 19, but, in any case the reduction is substantial. Furthermore, values at urban test sites are now in the same order of magnitude as rural test sites, which makes it more difficult to evaluate the effect of air pollution based on zinc corrosion.

When looking at Figure 20 and comparing the two last periods (1997–2000 and 2011–2014), there is a decrease in corrosion at all sites where a comparison is possible but it was not possible to capture this overall trend based on one-year data only (Figure 16, Figure 17 and Figure 18). Thus, the advantage of using four-year values for showing trends in zinc corrosion is quite evident. Four-year values are less sensitive to year-to-year variation in climatic parameters. The higher corrosion values also make it easier to identify significant trends for individual sites.

### 2.5. Trends in Copper Corrosion

**Figure 21:**
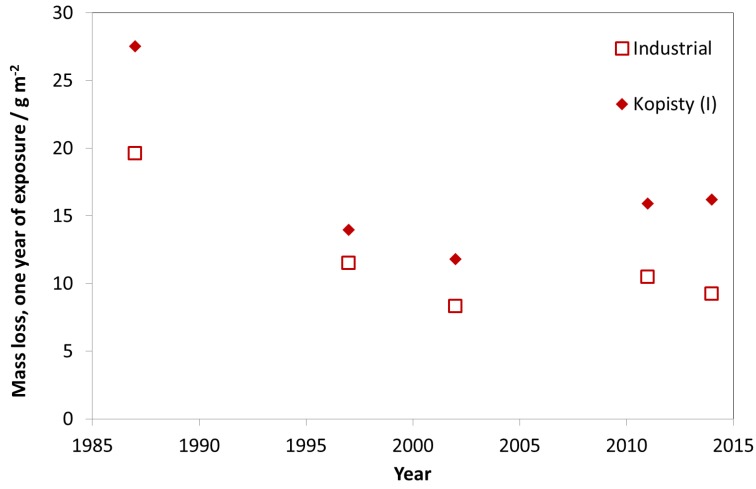
Copper corrosion at industrial sites and the industrial site Kopisty.

**Figure 22:**
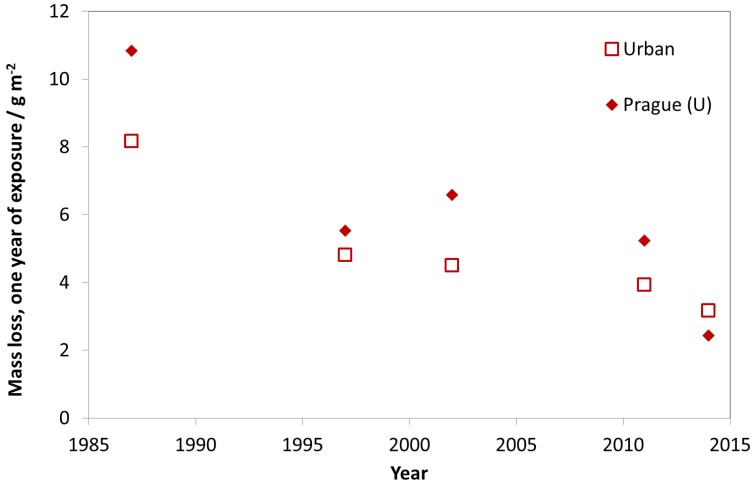
Copper corrosion at urban sites and the urban site Prague.

**Figure 23:**
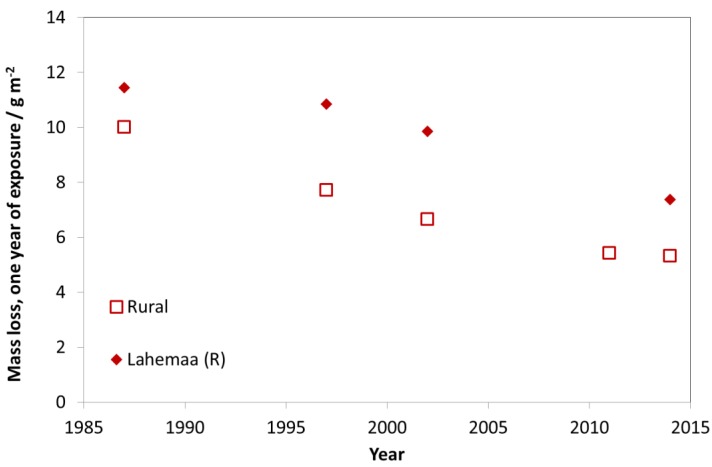
Copper corrosion at rural sites and the rural site Lahemaa.

Copper (Cu 99%, P 0.015%–0.04%) has been exposed for one year in 1987, 1997, 2002, 2011 and 2014 and the trends for industrial, urban and rural sites (accompanied with examples for individual sites) are presented in Figure 21, Figure 22 and Figure 23 in the same manner as for carbon steel (compare Figure 9, Figure 10 and Figure 11). The trends are similar for carbon steel and copper. After 1997, the decreasing trend is less evident, and, at the industrial sites, there are no decreasing trends at all.

**Figure 24:**
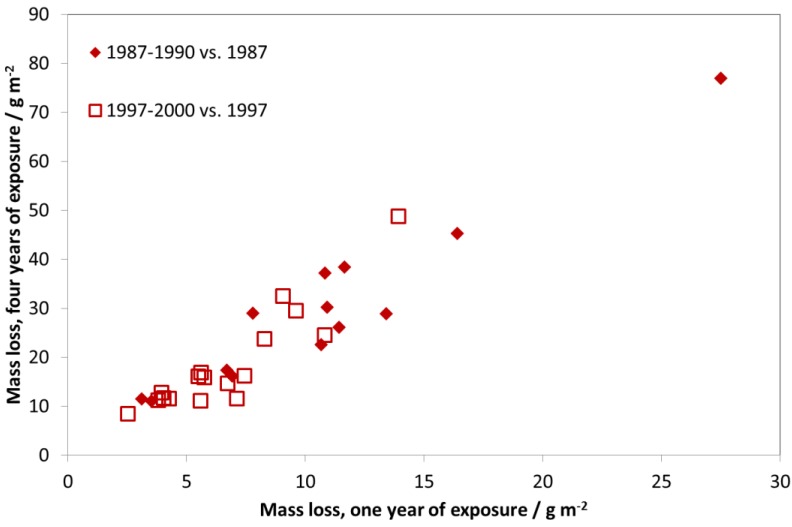
Copper corrosion one-year vs. four-year exposures for two different periods, 1987–1990 and 1997–2000.

**Figure 25:**
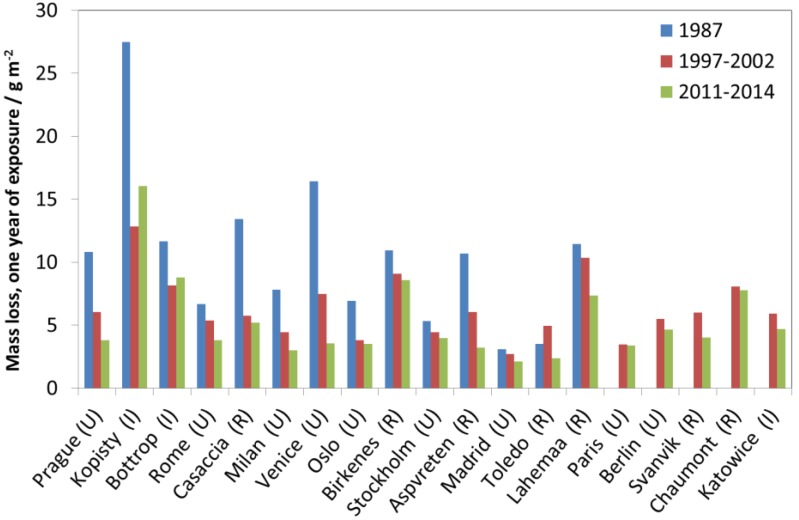
Copper corrosion at individual sites based on one-year data for 1987 and annual averages the periods 1997–2002 and 2011–2014.

Copper has been exposed for four years only for two exposure periods, 1987–1990 and 1997–2000. Figure 24 shows the mass loss after four years of exposure vs. mass loss after one year of exposure for the two periods. The relationship between one- and four-year data is about 1:3. Figure 25 summarises the trends in copper corrosion for all sites using three time periods, 1987, 1997–2002 average and 2011–2014 average, based on the available one-year data since four-year data are not available for the most recent period (2011–2014). The trends show decreasing corrosion for the most recent periods, except for Kopisty and Bottrop, both industrial sites.

### 2.6. Trends in Aluminium Corrosion

**Figure 26:**
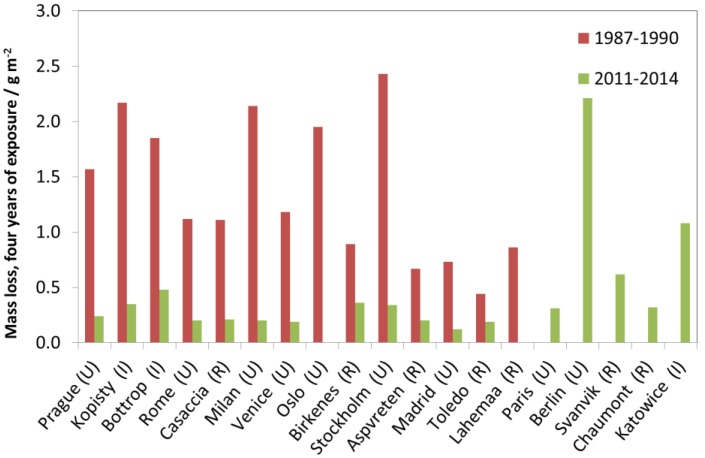
Aluminium corrosion: four-year exposures at individual sites for two exposure periods, 1987–1990 and 2011–2014.

For aluminium (>99.5%), data from the period 1987–1994 exist for two-, four- and eight-year exposures. In 2011, one set of aluminium samples was exposed with the intention to make a withdrawal after two years of exposure. Inspections at sites after two years indicated very low corrosion rates and therefore withdrawal was made after four years of exposure. For evaluation of trends in aluminium corrosion, there is thus only two four-year periods available, 1987–1990 and 2011–2014. The data are shown in Figure 26. The decrease in corrosion is substantial, but it should be noted that some of the new sites, especially Berlin, show corrosion values comparable to those obtained in 1987–1990.

### 2.7. Trends in Surface Recession of Limestone

**Figure 27:**
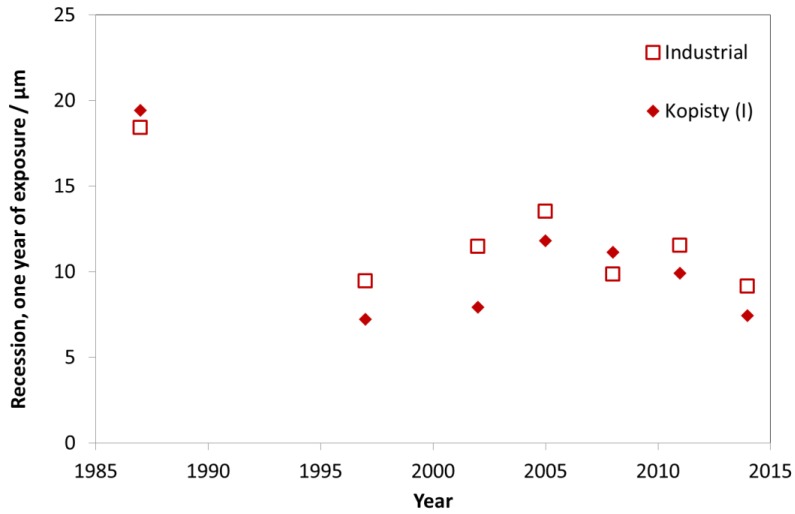
Limestone surface recession at industrial sites and the industrial site Kopisty.

**Figure 28:**
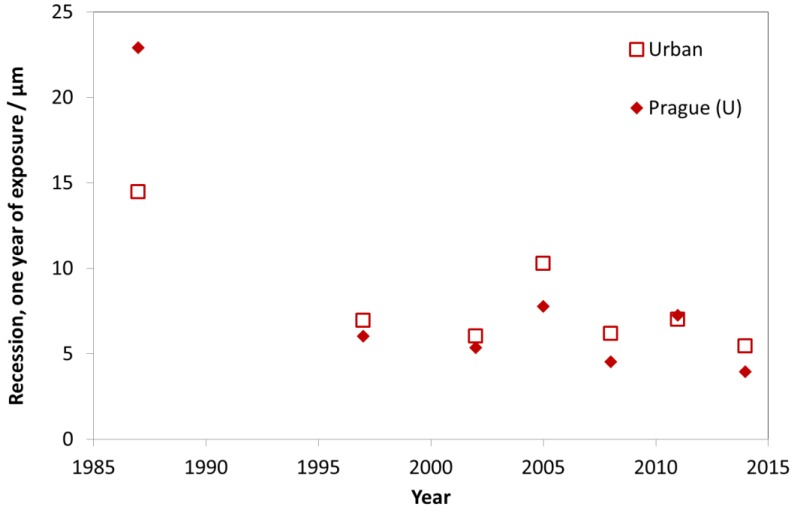
Limestone surface recession at urban sites and the urban site Prague.

**Figure 29:**
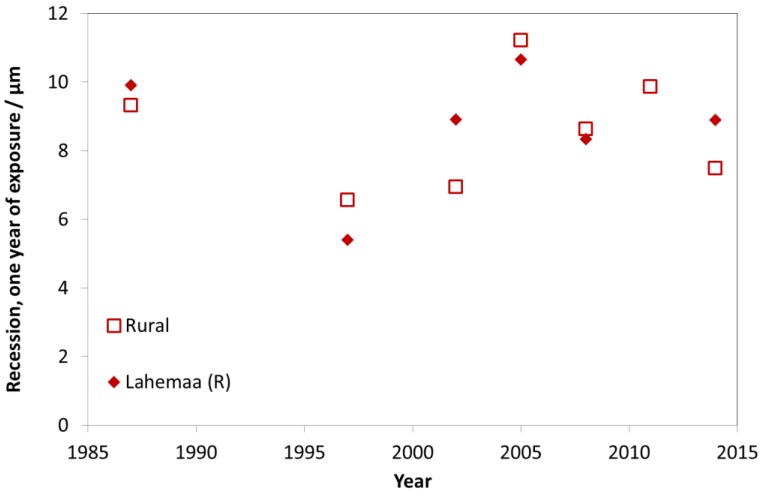
Limestone surface recession at rural sites and the rural site Lahemaa.

Figure 27, Figure 28 and Figure 29 show surface recession of limestone following the same model as for previous materials. Figure 27 shows industrial sites, Figure 28 urban sites and Figure 29 rural sites, each with one example site. The 1987 value is higher for industrial and urban sites but otherwise there is no evident decreasing trend after 1997, and the year-to-year fluctuations are substantial, indicating influence from varying climatic conditions.

**Figure 30:**
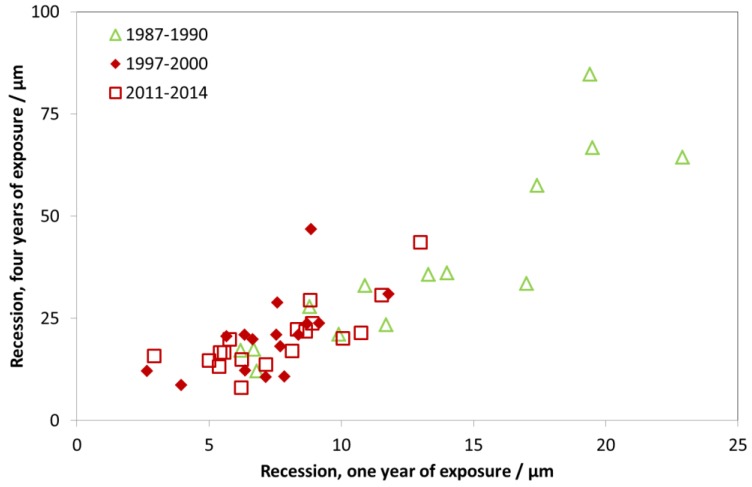
Limestone surface recession: one-year vs. four-year exposures for three different periods, 1987–1990, 1997–2000 and 2011–2014. The one-year corrosion values for comparison with 1987–1990 are taken from 1987, and for the other two periods calculated as averages of two one-year exposures for the years 1997/2002 and 2011/2014, respectively.

Figure 30 shows the four-year values vs. the one-year values, and shows almost a 1:4 correspondence, indicating linear kinetics, but with the one-year value being slightly higher, which is expected for limestone degradation.

**Figure 31:**
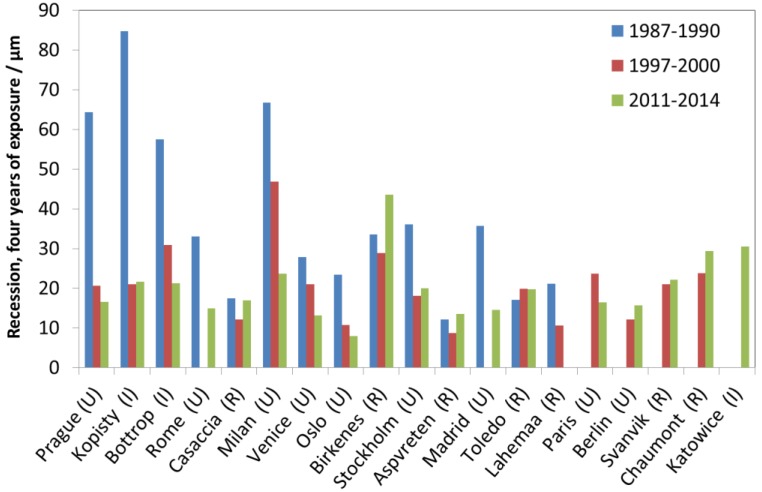
Limestone surface recession: four-year exposures at individual sites for three exposure periods, 1987–1990, 1997–2000 and 2011–2014.

Figure 31 shows all four-year data for limestone. Compared to other materials, the 1997–2000 to 2011–2014 trend is less evident, except for Prague, Bottrop, Milan, Venice and Oslo.

### 2.8. Trends in Soiling of Modern Glass

**Figure 32:**
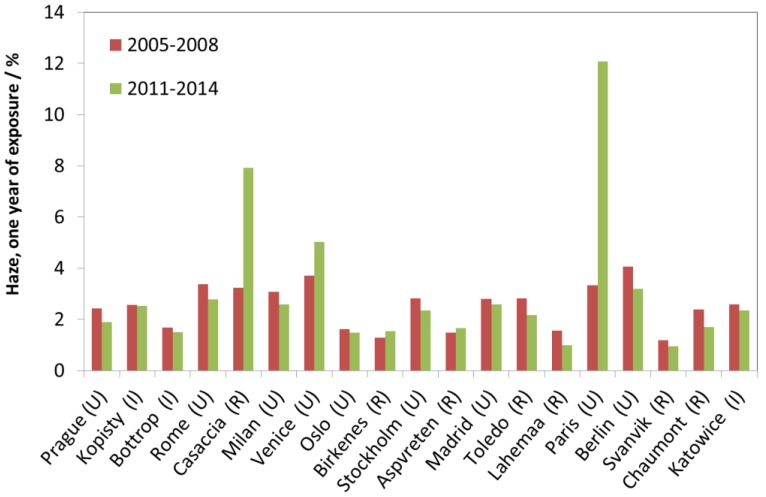
Soiling of modern glass after one-year of exposure, 2005–2008 and 2011–2014, in sheltered position expressed as haze (%).

Four one-year exposures of glass in sheltered conditions have been carried out between 2005 and 2014. Of the materials presented here, this is the only material exposed in sheltered position. The soiling is quantified using the haze parameter, which is the ratio between the diffuse and direct transmitted light [10]. Figure 32 shows that the haze increases, moderately for Birkenes and Aspvreten, and strongly for Casaccia, Venice and Paris. A sharp increase is difficult to explain for the site Casaccia, but is caused by the moving of the site for Paris in 2011. Haze is relatively constant or decreasing for the other sites.

## 3. Discussion

The results presented show that corrosion and pollution have decreased significantly during the period where data are available (1987–2014). When looking into the details of the decreasing trends, a shift in the magnitude generally occurs around 1997; a sharp decrease changed to a more modest decrease or to a constant level without any decrease. The levels of the pollutant SO_2_ and the corrosion of the materials carbon steel and copper have decreased even after 1997, more pronounced in urban areas, while the other materials show no decreases in corrosion or soiling after 1997, when looking at one-year values. When looking at four-year values, however, there is a significant decrease after 1997 for zinc, which is not evident when looking at the one-year values. The advantage of using four-year values for showing trends in corrosion is that it is less sensitive to year-to-year variation in climatic parameters (temperature, relative humidity, and precipitation). The higher corrosion values make it also easier to identify significant trends for individual sites.

The reduction in corrosion has changed the way we look at “industrial”, “urban” and “rural” sites from a corrosion point of view. In the past, these labels could in some way relate to the level of corrosion, but this is no longer the case, especially for some materials. The labels may be useful for indicating the type of pollution (dominated by SO_2_, NO_2_ or O_3_, for example) but they are not useful at all for classifying levels of corrosivity in a quantitative way.

ISO 9223 provides a system for classification of corrosivity. When applying this to the data, the change in corrosion is often from C4 to C3 or even C2. This has practical implications, as it is expected that lifetimes of constructions affected by corrosion is significantly prolonged in some atmospheres. However, there are contemporary environments, such as the Berlin site (Figure 26), which still show corrosion values corresponding to past levels. This is a new type of site in the ICP Materials programme, close to the road with high levels of particulate deposition. This illustrated the need for continuous measurements of pollutants and the awareness to possible new or reappearing emission sources.

The paper also presents results on corrosion kinetics by comparison of one- and four-year corrosion values. Some materials, such as carbon steel and copper, show kinetics relatively independent of sites, while other materials, especially zinc, show substantial variation in kinetics for the first four years, which needs to be taken into account when producing new and possibly improved models for corrosion.

ICP Materials is now in the process of starting a new exposure (2017 and 2017–2020), which will provide new sets of one- and four-year data. Included in the programme will be, in addition to earlier exposures described in this paper, corrosion of stainless steel and soiling of two coil coated materials (white and brown) as well as soiling of two stone materials (limestone and marble). These additions show a direction of future development of the programme, i.e. more focused on soiling of materials and the effect of particulate matter.

## 4. Materials and Methods

Procedures used in ICP Materials are described in the technical manual, which includes information on materials, environment, how to run a test site (29 pages) and detailed descriptions of all test sites (86 pages). It is beyond the scope of this paper to repeat all this information, but, in general, exposures conform to procedures described in ISO 8565. The technical manual and all reports produced by ICP Materials are available for download at the ICP Materials home page.

In addition, all data discussed and presented in this paper can be found at the Supplementary File. They are also available for download, submitted as open access, at the ICP Materials home page.

## Supplementary Materials

The following are available online at http://www.mdpi.com/1996-1944/10/8/969/s1.
